# Detecting Hexafluoroisopropanol Using Soft Chemical Ionization Mass Spectrometry and Analytical Applications to Exhaled Breath

**DOI:** 10.1021/jasms.3c00042

**Published:** 2023-03-30

**Authors:** Florentin Weiss, Anesu Chawaguta, Matthias Tolpeit, Valeria Volk, Arne Schiller, Veronika Ruzsanyi, Petra Hillinger, Wolfgang Lederer, Tilmann D. Märk, Chris A. Mayhew

**Affiliations:** † Institute for Breath Research, Universität Innsbruck, Innrain 66, A-6020 Innsbruck, Austria; ‡ Department of Anaesthesiology and Critical Care, Medical University of Innsbruck, Anichstraße 35, A-6020 Innsbruck, Austria; § Institute for Ion Physics and Applied Physics, Universität Innsbruck, Technikerstraße 25/3, A-6020 Innsbruck, Austria

## Abstract

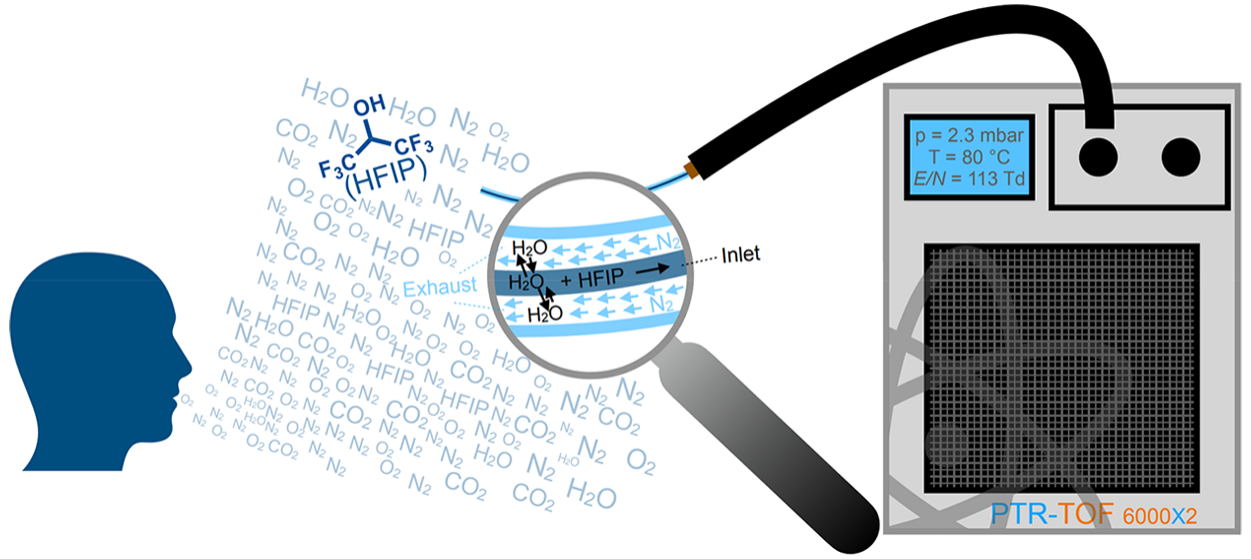

Here we explore the potential use of proton transfer reaction/selective reagent ion-time-of-flight-mass spectrometry (PTR/SRI-ToF-MS) to monitor hexafluoroisopropanol (HFIP) in breath. Investigations of the reagent ions H_3_O^+^, NO^+^, and O_2_^+•^ are reported using *dry* (relative humidity (rH) ≈ 0%) and *humid* (rH ≈ 100%)) nitrogen gas containing traces of HFIP, i.e., divorced from the complex chemical environment of exhaled breath. HFIP shows no observable reaction with H_3_O^+^ and NO^+^, but it does react efficiently with O_2_^+•^ via dissociative charge transfer resulting in CHF_2_^+^, CF_3_^+^, C_2_HF_2_O^+^, and C_2_H_2_F_3_O^+^. A minor competing hydride abstraction channel results in C_3_HF_6_O^+^ + HO_2_^•^ and, following an elimination of HF, C_3_F_5_O^+^. There are two issues associated with the use of the three dominant product ions of HFIP, CHF_2_^+^, CF_3_^+^, and C_2_H_2_F_3_O^+^, to monitor it in breath. One is that CHF_2_^+^ and CF_3_^+^ also result from the reaction of O_2_^+•^ with the more abundant sevoflurane. The second is the facile reaction of these product ions with water, which reduces analytical sensitivity to detect HFIP in humid breath. To overcome the first issue, C_2_H_2_F_3_O^+^ is the ion marker for HFIP. The second issue is surmounted by using a Nafion tube to reduce the breath sample’s humidity prior to its introduction into drift tube. The success of this approach is illustrated by comparing the product ion signals either in *dry* or *humid* nitrogen gas flows and with or without the use of the Nafion tube, and practically from the analysis of a postoperative exhaled breath sample from a patient volunteer.

## 1 Introduction

Sevoflurane (1,1,1,3,3,3-hexafluoro-2-(fluoromethoxy)propane, CH_2_FOCH(CF_3_)_2_, molar mass 200.055 g/mol)) is a commonly used inhalation anesthetic to induce and maintain general anesthesia. Although it is predominantly eliminated from the body via exhalation, a small loss (≈ 3 to 6%) results from its metabolism predominantly in the liver by the cytochrome P450 2E1 (CY2PE1) to inorganic fluoride and the volatile organic compound hexafluoroisopropanol (hexafluoro-2-propanol, (CF_3_)_2_CHOH), HFIP, molar mass 168.05 g/mol).^1,2^ Using gas-chromatography–mass spectrometry, Ghimenti et al. investigated the postoperative elimination of sevoflurane and HFIP of six critically ill patients following their surgeries.^3^ They demonstrated that the unconjugated fraction of HFIP (<15% of the total HFIP concentration in the blood^1^) could be detected in exhaled breath samples and hence suggested that the monitoring of sevoflurane and its metabolite could potentially be used in a breath test to noninvasively assess liver function. A particularly novel application proposed by Ghimenti et al. is the use of a breath test to assess liver function following a liver transplant by monitoring the washouts of sevoflurane and HFIP. This could possibly be used to eliminate the need for invasive procedures such as liver biopsies that are commonly used to determine postoperative recovery following liver transplant. Toward these goals, Ghimenti et al. presented pharmacokinetic models of the postoperative elimination of sevoflurane and HFIP. An earlier study by King et al. also modeled sevoflurane in exhaled breath for estimating physiological processes.^4^ King et al. explored some potential applications of breath analysis in anesthesia, including the real-time assessments of the anesthetic state through the measurements of inhalation anesthetic concentrations in exhaled breath.

Given the potential clinical use for monitoring HFIP and sevoflurane in exhaled breath, and given our recent proton transfer reaction-time-of-flight-mass spectrometry (PTR-ToF-MS) and selective reagent ion-time-of-flight-mass spectrometry (SRI-ToF-MS) studies of the reactions of H_3_O^+^ and O_2_^+•^, respectively, with sevoflurane,^5,6^ we report here the first corresponding measurements for HFIP. In this paper, details on the primary product ions as a function of the reduced electric field and their dependence on the humidity of the nitrogen carrier gas being used to introduce trace quantities of HFIP into the drift tube are reported. (The reduced electric field is the ratio of the electric field strength (*E*) to the total molecular number density (*N*) in the drift (reaction) tube of the PTR/SRI-ToF-MS. The commonly used unit for *E*/*N* is the Townsend (Td), where 1 Td = 1 × 10^–17^ V cm^2^.)

Humidity-dependent measurements are important because of potential secondary reactions of volatile’s primary product ions with any water vapor present in the drift tube of the PTR/SRI-ToF-MS. These secondary reactions were found to be significant for the primary ions produced from the reactions of both H_3_O^+^ and O_2_^+•^ with sevoflurane (and other fluranes)^5−7^ and agree with an earlier selected ion flow tube-mass spectrometry (SIFT-MS) investigation of sevoflurane by Wang et al.^8^ Similar problems with the product ions resulting from HFIP may also occur, which, if they do, would significantly diminish their concentrations and in turn reduce the PTR/SRI-ToF-MS’s analytical sensitivity for monitoring HFIP in exhaled breath.

A key aim of this study is to present investigations of two approaches that independently and together significantly increase the yields of the product ions of HFIP and hence improve the analytical sensitivity of the PTR/SRI-ToF-MS for this compound. The first approach involves operating the PTR/SRI-ToF-MS with its ion funnel activated (this will be referred to in this paper as RF-mode), which increases the transmission of both the reagent and product ions into the transfer optics and hence into the ToF-MS. The second approach is the inclusion of a Nafion tube to form part of the heated inlet carrier gas/sample line, which is connected to the inlet of the instrument’s drift tube. Much of the water in an analytical gas sample should be removed as it passes through the Nafion tube. Hence, we can expect significantly improved intensities of those primary product ions that have facile reactions with water. A secondary aim of this work is to illustrate the analytical potential of PTR/SRI-ToF-MS to quantify and monitor both sevoflurane and HFIP elimination from the human body by analyzing the exhaled breath of a patient following surgery.

The primary goal of this paper is to present for the first time the potential analytical benefits of using a Nafion tube for use in analyzing trace volatiles in *humid* gas samples using PTR/SRI-ToF-MS. However, the work presented also has implications to other areas of application. The possible development of volatile probes in exhaled breath to determine liver function, especially following transplant surgery, has already been mentioned above. Other uses include monitoring the postoperative recovery of patients, determining the washout of the inhalation anesthetics from the body, and determining the workplace exposure of hospital staff in the postoperative observation units to inhalation anesthetics, the latter of which has been investigated by Rieder et al.,^9,10^ and extended in a more detailed higher mass resolution proton transfer reaction-mass spectrometric study by Trefz et al.^11^

## 2 Materials and Methods

### 2.1 Proton Transfer Reaction-Time-of-Flight-Mass Spectrometer/Selective Reagent Ionization-Time-of-Flight-Mass Spectrometer

A SRI-ToF mass spectrometer is simply a PTR-ToF-MS instrument operating with any reagent ion other than H_3_O^+^. The most popular reagent ions used in the field of analytical chemistry are NH_4_^+^,^12^ NO^+^, and O_2_^+•^.^13^ In this paper, we have used H_3_O^+^ (PTR-mode), and NO^+^ and O_2_^+•^ (SRI-mode). The operational procedures of PTR-ToF-MS and its adaptation to SRI operation have been discussed in detail elsewhere.^14−20^ Furthermore, the methods and the specific instrumentation utilized in this present work are similar to those described in considerable detail in a recent publication dealing with inhalation anesthetics.^6^ Therefore, the present work will only include a brief overview and will focus on details that go beyond the previous study.

The PTR/SRI-ToF mass spectrometer used in this study is a state-of-the-art compact high-performance extended PTR-TOF 6000 X2 (Ionicon Analytik GmbH, Austria).^6,12,21−23^ This instrument has a mass resolving power (*m*/Δ*m*) of approximately 6000 at *m*/*z* 147 and a typical analytical sensitivity of approximately 2000 counts per second per part per billion by volume of a trace volatile (cps/ppb_v_).

The drift tube of the PTR-TOF 6000 X2 is equipped with a radio frequency (RF) ion funnel, which is contained within the end section (last 1.7 cm) of the drift tube. The drift tube has a total length of 8.6 cm. When operational (RF-mode), this ion funnel improves the transmission of both the reagent and product ions from the drift tube into the detection region of the instrument by helping to focus the ions into the central region of the drift tube. In this study, the instrument was used both *without* (DC-mode) and *with* (RF-mode) the funnel operational. Independent of its operational mode, the drift tube was maintained at a temperature of 353 K and kept at a constant pressure of either 2.6 mbar when using H_3_O^+^ as the primary reagent ion or 2.3 mbar when using NO^+^ or O_2_^+•^ as reagent ions.

In DC-mode, an *E*/*N* value is well-defined throughout the whole of the drift tube. For the DC-mode measurements, the *E*/*N* range applied (65–205 Td) was scanned in steps of 10 Td by simply changing the drift tube voltage by appropriate amounts while keeping *N* constant, with mass spectra data being collected at each *E*/*N* value for 10 s. The product ion peak positions and their areas were then determined. Each *E*/*N* measurement was repeated three times. The three values obtained for the areas (ion signals) and peak positions (*m*/*z* values) for each *E*/*N* value were averaged with the mean being provided.

When the ion funnel is operational (RF-mode), an *E*/*N* value can only be accurately defined for the first 6.9 cm of the drift tube. In order to still be able to compare measurements between DC- and RF-modes, the operational parameters of the ion funnel were so adjusted that no additional fragmentation occurred at a specified *E*/*N* value used in the front end (first 6.9 cm) of the drift tube. For example, to achieve the same HFIP product ion branching percentages determined in DC-mode at an *E*/*N* of 113 Td (the reason for choosing this *E*/*N* value will become apparent in the results section), the RF operational parameters were tuned to the following values: the RF amplitude (peak to peak) was set to 35 V, the RF frequency was maintained at 1 MHz, and the last drift ring and the sampler cone voltages were set to 10 V.

Use of a high purity *dry* nitrogen (99.9999%, < 3 ppm_v_ water) carrier gas for the sample and hence the buffer gas in the drift tube resulted in what we shall refer to as *dry* drift tube conditions. Note that the humidity in the drift tube will be higher than this owing to residue water vapor being present in the drift tube. To achieve a higher relative humidity (rH) in the drift tube, namely a rH ≈ 100%, the nitrogen gas was bubbled through distilled water kept at room temperature prior to its introduction into the drift tube. We will refer to this as *humid* drift tube conditions.

### 2.2 Reagent Ion Production

The reagent ions H_3_O^+^, NO^+^, or O_2_^+•^ were created by introducing water vapor, nitrogen/oxygen mix (3:1), or oxygen into the hollow cathode ionization source, as has already been described in detail elsewhere.^16,18,24^ As will be discussed in the Results section, H_3_O^+^ and NO^+^ show no observable reaction with HFIP. Thus, for this study, O_2_^+•^ is the most important reagent ion. Therefore, it needs to be pointed out that in addition to O_2_^+•^, other (impurity) reagent ions are also produced in the hollow cathode ionization source. These result from residual water in the hollow cathode and from the back diffusion of nitrogen/air and water from the drift tube. The dominant impurity reagent ions are found to be H_3_O^+^, H_3_O^+^·H_2_O, NO^+^, and NO_2_^+^.^6^ Their combined contribution to the total reagent ion signal is less than 10% under *dry* drift tube conditions for all *E*/*N* values, and less than 10% and 30% at the highest and lowest *E*/*N* values used, respectively, under *humid* drift tube conditions.^6^ For *humid* drift tube conditions at about 65 Td (the lowest *E*/*N* value investigated), the major reagent ions were found to be H_3_O^+^·H_2_O (∼15%) and H_3_O^+^ (∼10%). Given that H_3_O^+^, H_3_O^+^·H_2_O, and NO^+^ do not show any significant reaction with HFIP and given that the NO_2_^+^ impurity ions contribute less than about 2% to the total reagent ion signal intensity at any given *E*/*N* value for any humidity level in the drift tube, the effects of the impurity reagent ions present in the drift tube on the product ion distributions can be ignored.

### 2.3 SRI-ToF-MS Data Analysis

Data are generally provided in terms of the raw product ion signals in units of cps. However, to help interpret the results, it is useful to take into account any changes in the O_2_^+•^ reagent ion signals as *E*/*N* is changed and in particular when comparing data obtained under *dry* drift tube conditions to those taken under *humid* drift tube conditions.^5,6^ For example, compared to the reagent ion signal under *dry* drift tube condition, the O_2_^+•^ intensity is reduced by about 50% when operating the drift tube under *humid* conditions at the lowest *E*/*N* value used.^6^ To account for the changes in the O_2_^+•^ intensity, the product ion signals are normalized to a constant reagent ion signal, which results in the product ion signals being given in units of normalized counts per second (ncps). In this study we have normalized the product ion yields to 2 × 10^6^ O_2_^+•^ reagent ions per second. This value was chosen because it is close to the maximum O_2_^+•^ cps recorded. Note that the ^16^O^16^O^+•^ signal intensity at *m*/*z* 31.990 could not be measured directly, owing to the high amount of ions saturating the detector. Instead, the signal intensity for ^16^O^18^O^+•^ at *m*/*z* 33.994 was determined, from which the ion intensity ^16^O^16^O^+•^ was determined and used in the normalization procedure.

### 2.4 Gas Chromatography-Quadrupole-Time-of-Flight Mass Spectrometry (GC-qToF-MS) Measurement of HFIP

Given that charge transfer is the dominant reaction mechanism (see the Results section), to further investigate the product ions that result from the transfer of an electron from the highest occupied molecular orbital (HOMO) of HFIP, we recorded a high mass resolving power (*m/Δm* ≈ 40000) 70 eV electron impact mass spectrum (EI-MS) of HFIP. For this, we used a recently purchased 7250GC-qToF (Agilent Technologies, USA). To provide traces of HFIP, a needle tip of a 5 μL syringe (Hamilton Company, USA) was simply dipped into cooled liquid HFIP. Traces of the substance that were present on the surface of the needle tip were then drawn into the GC column during the direct column injection process and then analyzed using the qToF mass spectrometer.

Consideration of the differences in the internal energy generated in the singly charged parent of a volatile following the loss of an electron via a 70 eV electron ionization or via the lower energy electron transfer process means that care must be taken when comparing the two mass spectra generated. Nevertheless, the EI-MS mass spectrum does provide useful information to help confirm which primary product ions observed result from the electron transfer. Charge (electron) transfer processes are discussed in detail by Jarvis et al.,^25^ and in particular short-range (intimate contact) ion–molecule processes are emphasized that lead to product ions resulting from charge transfer in competition to those that can only be produced if atoms are transferred between the reactants.

### 2.5 Use of a Nafion Tube to Reduce the Humidity of the Sample Gas Prior to its Introduction into the Drift Tube

In SRI-ToF-MS investigations dealing with the reactions of O_2_^+•^ with sevoflurane,^6^ it was found that the primary product ions reacted efficiently with the water vapor present in the drift tube, leading to a significant decrease in their intensities, especially at the lowest *E*/*N* values investigated. While these secondary product ion–water processes are not a problem for detecting sevoflurane, owing to its extremely high concentrations in exhaled breath (even days after surgery), they could be a problem for detecting the much lower breath concentrations expected for HFIP.^3^

For any breath volatile whose primary product ions react with water, it is useful to remove as much of the water vapor from the breath sample as possible prior to introducing it into the reaction region of the PTR-TOF 6000 X2, while at the same time not losing too much of the volatile under investigation. To achieve this, a Nafion tube can be inserted into the gas/sample inlet line leading to the inlet of the drift tube to deliver flows with lower humidities at its output.^26^ Leckrone and Hayes explain that the resulting gradient in the water vapor pressure across the Nafion membrane drives the transfer of water from the carrier-gas flow inside the tube to the purge-gas flow outside of the tube.^26^ Nafion tubing has been successfully applied to GC studies involving trace volatile organic compounds,^26−29^ but to our knowledge not to SRI-ToF-MS studies.

In this work, we utilized a Perma Pure drier PPMD-070–72-F Nafion tube of 60 cm length and internal/outside diameters of 1.52 mm/1.83 mm, respectively. This Nafion tube was completely enclosed inside a 1/4 in. PTFE/PVDF tube that was continuously purged by a counter flow of *dry* nitrogen at a flow rate of 300 mL/min throughout the measurements. The Nafion and PTFE/PVDF tubes were placed inside an oven to maintain a constant temperature of 313 K because permeation of water through Nafion is temperature dependent. The outlet of the Nafion tube was connected to the inlet gas line of the PTR-TOF 6000 X2, which was itself heated and maintained at 353 K.

### 2.6 Chemicals, Preparation of HFIP Gas Standards, and Instrumental Calibration

HFIP (CAS: 920–66–1) was purchased from Sigma-Aldrich with a stated purity of ≥99.8%. It was used without further purification.

To determine the primary product ions resulting from the reaction of O_2_^+•^ with HFIP, gas standards with a HFIP volume-mixing ratio of 1.05 ppm_v_ in nitrogen were prepared using a method previously described in detail for our SRI-ToF-MS measurements with sevoflurane.^6^ Separate *E*/*N* sweeps (as described in section 2.1) for three samples containing 1.05 ppm_v_ HFIP in *dry* nitrogen were performed first in order to unambiguously identify the different product ions formed.

Before attempting the detection of HFIP in breath samples, it was essential to determine the effects of sample humidity on the primary product ions independently from the complex chemical environment of *humid* exhaled breath. We therefore conducted test measurements using three *dry* and three *humid* gas standards containing 1.05 ppm_v_ HFIP in nitrogen gas at a rH ≈ 0% and a rH ≈ 100%, respectively. For all of these measurements, the drift tube of the PTR-6000 X2 was operated in DC-mode using an *E*/*N* value of 113 Td.

Sevoflurane (Sevorane, CAS: 28523–86–6) was purchased from AbbVie GmbH (Vienna, Austria). In order to determine the levels of sevoflurane and HFIP present in the clinical samples of exhaled human breath (see section 2.7), it is necessary to convert the counts per second signals of the primary product ions provided to volume mixing ratios (in ppb_v_), which requires calibrating the analytical instrument using the same operational parameters as for the measurements of the breath and room air samples. For sevoflurane, we used gas standards containing 2, 4, 8, 16, 32, 63, 127, and 254 ppb_v_ sevoflurane (in 0% rH nitrogen) with the drift tube operating in DC-mode at 147 Td. Additional sevoflurane calibration standards (16, 32, 63, 127, 254, and 507 ppb_v_) were prepared using 20% rH nitrogen to permit the breath samples taken after 21.25 and 27.25 h (dilution factor 5) and 45.25 and 52.25 h (dilution factor 4) after anesthesia to be given in ppb_v_, which takes into account possible losses due to humidity. For the HFIP calibration, gas standards containing 106, 212, 424, and 844 ppb_v_ of HFIP in *humid* nitrogen were prepared. These gas standards were measured under the same experimental conditions as those used for the analysis of the breath sample, namely using RF-mode at an effective *E*/*N* value of 113 Td and, for reasons given below in section 2.7, at 147 Td, and with the use of a Nafion membrane filter tube that had been installed in the inlet line.

In order to characterize possible interferences of large sevoflurane concentrations on the *m*/*z* values of the HFIP product ions, we performed additional measurements using samples of *humid* nitrogen containing approximately 500 ppm_v_ sevoflurane to simulate conditions encountered in breath samples of patients shortly after surgery. These samples were analyzed in the same way as the patient breath samples described above for the HFIP measurements, namely RF-mode and use of Nafion membrane filter.

### 2.7 Patient’s Details and Method for the Collection of Exhaled Breath

To illustrate the potential for detecting trace amounts of HFIP in exhaled breath and to demonstrate the usefulness of our analytical method, a breath sample was collected from one volunteer patient following a surgical procedure for which sevoflurane was used as the inhalation anesthetic. This volunteer was an 18-year-old male with a body mass index of 22.6 kg/m^2^ who received surgery as a treatment for chronical pilonidal disease. The patient’s preanesthesia medical comorbidities were categorized as mild systemic disease (American Society of Anaesthesiologists (ASA) physical status classification ASA 2). General anesthesia was induced with 180 mg of propofol (1%) and 0.35 mg of fentanyl. Following muscle relaxation with 30 mg of rocuronium bromide, anesthesia was maintained with sevoflurane at 2 vol % and continuous infusion of 0.18 mg of remifentanil delivered to the patient by vaporizer. Antimicrobial treatment with 500 mg of metronidazole and 1500 mg of cefuroxime and 793 mL of crystalloid infusion was administered. The total duration of anesthesia from induction to emergence was 91 min, with the surgery core time of approximately 60 min.

Samples of end-tidal phase exhaled breath were taken from the patient, with the end-tidal (alveolar) phase being determined by monitoring the CO_2_ levels with an infrared sensor. The breath and room air samples were collected in the hospital at approximately 1.25 h before the start of the anesthesia and then after surgery at 0.5, 5.25, 21.25, 27.25, 45.25, and 52.25 h, with time zero being defined as the patient’s emergence from the anesthesia. The first breath and room air samples after surgery were collected in the recovery room. All other breath and room air samples from the volunteer were collected in the patient’s private hospital room using 250 mL glass syringes (Socorex). The analytical methods employed to analyze the end-tidal breath and room air samples were identical to those described in detail in a previous study.^6^ In brief, the breath and room air samples were brought to the laboratory of the Institute for Breath Research within 1 h of their collection. They were then analyzed using the PTR-TOF 6000 X2 with the drift tube operating in RF-mode at an effective *E*/*N* value of 113 Td to maximize analytical sensitivity and the use of the Nafion filter for the HFIP determination (using undiluted samples). DC-mode at 147 Td was used to quantify sevoflurane in the breath samples, which had to be diluted to avoid saturation of the product ions. This had an added benefit of reducing the samples’ humidities prior to their introduction into the drift tube. The dilution factors, given in the chronological order of the measurements, were 4, 62500, 100, 5, 5, 4, and 4.

Owing to inadvertently forgetting to reset the SRI-ToF-MS drift tube operational parameters from a previous measurement, three out of seven breath sample and room air measurements to determine the HFIP concentrations, namely the samples collected before surgery and at 21.25 and 45.25 h after emergence from anesthesia, were measured at an *E*/*N* of 147 Td. We therefore had to perform a separate calibration with the drift tube *E/*N set to 147 Td using the same method described in section 2.6.

## 3 Results

### 3.1 H_3_O^+^ Reagent Ion Measurements with HFIP

No significant reaction of H_3_O^+^ with HFIP was observed. This was initially surprising given the reported values of the gas phase basicities (GBs) of H_2_O and HFIP being 660.0 kJ mol^–1^ and 656.2 kJ mol^–1^, respectively.^30^ Using these values, the reaction of H_3_O^+^ with HFIP leading to HFIP·H^+^ and H_2_O is only endoergic by Δ*G* = 3.8 kJ mol^–1^, where Δ*G* is the change in the free energy. This is close to thermoneutral, which would be easily overcome within the drift tube of the SRI-ToF-MS through the suprathermal translational energies of the reagent ions. Exoergic reactions are observed to always proceed at the theoretical collisional rate, i.e., at the upper limit for ion–molecule reactions so that proton transfer will occur for every gas-kinetic collision, as highlighted by Böhme et al.^31^ Even those proton transfer reactions close to thermoneutral will still be facile. Hence, if it occurs, a proton transfer reaction should be observed, especially if dissociative proton transfer occurs. For example, a review of its structure implies that upon protonation the elimination of H_2_O from HFIP·H^+^ could be expected to be facile. Even if the reaction is nondissociative, so that a back reaction with the residual H_2_O in the drift tube occurs, thereby reducing the sensitivity for HFIP·H^+^ detection, we should still expect to see a signal associated with the protonated parent, especially when using a *dry* inlet sample nitrogen gas that is being introduced into the drift tube and given the high HFIP concentration that is added to this carrier gas.

When proton transfer is endoergic (Δ*G* > 0), the associated reaction rate coefficient (*k*_exp_) is found to be related to the collisional rate coefficient (*k*_c_) according to *k*_exp_ = *k*_c_ × exp(−Δ*G*/*N*_A_*k*_B_*T*),^31^ where *k*_B_ is Boltzmann’s constant, *N*_A_ is Avogadro’s number, and *T* is the thermodynamic temperature. That no reaction is observed suggests that the Δ*G* for the transfer of a proton from H_3_O^+^ with HFIP is significantly positive, in turn suggesting that the GB value of HFIP available in the literature is possibly incorrect. We therefore undertook density functional theory calculations to determine the GBs of water and HFIP. For these, the Gaussian09W program with the GaussView05 for Windows interface and the B3LYP functional with the 6-31+G(d,p) basis set at 298 K was used.^32^ The values obtained are GB(H_2_O) = 653 kJ mol^–1^ and GB(HFIP) = 623 kJ mol^–1^. The calculated GB(H_2_O) is in good agreement with the accepted value given above, and the use of difference in the calculated GBs will be even more accurate. Thus, according to these calculated values, proton transfer from H_3_O^+^ to HFIP at 298 K is in fact not thermoneutral as suggested by the value above but highly endoergic with a Δ*G* = +30 kJ mol^–1^. Although, the temperature of the drift tube in this study is at 353 K and the translational temperature of the ions is even higher owing to kinetic energy gained by the reagent ions in the electric field, the Δ*G* calculated at 298 K still explains why no significant reaction of H_3_O^+^ with HFIP is observed even in the higher effective temperatures in the drift tube. It was this lack of reaction that in fact led us to undertake a HFIP study involving other reagent ions, namely the reactions of NO^+^ and O_2_^+•^, the results for which are discussed in sections 3.2 and 3.3, respectively.

### 3.2 NO^+^ Reagent Ion Measurements with HFIP

The ionization energy of HFIP is reported to be 11.94 eV,^33^ whereas the recombination energy of NO^+^ is 9.26 eV.^34^ Therefore, electron transfer from HFIP to NO^+^ leading to the products HFIP^+•^ and NO is endothermic by 2.68 eV (259 kJ mol^–1^) and hence cannot occur. However, reactions of NO^+^ with volatiles can occur via other pathways. These include abstraction of a hydride ion (H^–^) from the analyte or an association process.^24,35^ Nevertheless, none of these reaction pathways were observed at any *E*/*N* value investigated. HFIP is therefore found to be unreactive to NO^+^.

### 3.3 O_2_^+•^ Reagent Ion Measurements with HFIP

**Figure 1:**
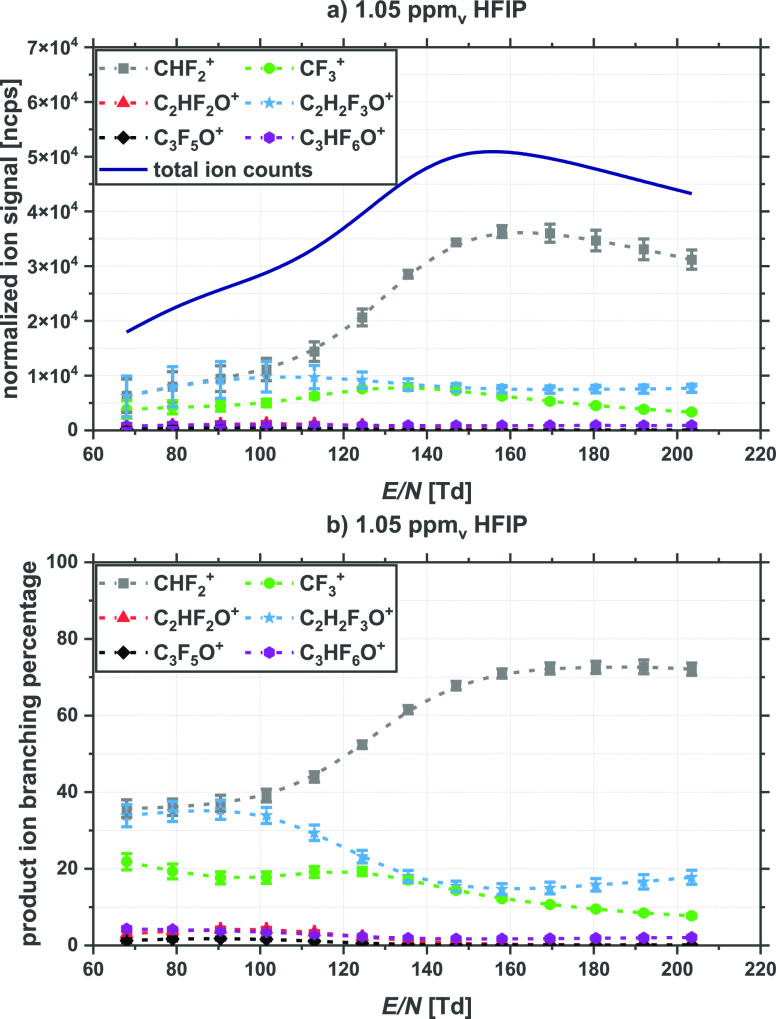
Product ion signals resulting from the reactions of O_2_^+•^ with HFIP (volume mixing ratio of 1.05 ppm_v_) in a dry nitrogen buffer gas, and hence dry drift tube conditions, as a function of the reduced electric field (*E*/*N*) in Townsend (1 Td = 10^–17^ V cm^2^) given in (a) normalized counts per second (ncps) and (b) branching percentages. The drift tube was operated in DC-mode.

Unlike the other two reagent ions, O_2_^+•^ is found to react efficiently with HFIP resulting in six product ions. In order of increasing *m*/*z* (lightest isotopomer), these are CHF_2_^+^ (*m*/*z* 51.005), CF_3_^+^ (*m*/*z* 68.995), C_2_HF_2_O^+^ (*m*/*z* 78.999), C_2_H_2_F_3_O^+^ (*m*/*z* 99.006), C_3_F_5_O^+^ (*m*/*z* 146.987), and C_3_HF_6_O^+^ (*m*/*z* 166.993). Figure 1 presents a graphical representation of the product ion distributions as a function of *E*/*N* in terms of (a) ncps and (b) product ion branching percentages. CHF_2_^+^ is found to be the dominant product ion throughout the *E*/*N* range investigated, with a maximum intensity occurring at approximately 160 Td, followed in intensity by the product ions CF_3_^+^ and C_2_H_2_F_3_O^+^, with maxima at approximately 130 and 113 Td, respectively. The remaining three product ions C_2_HF_2_O^+^, C_3_F_5_O^+^, and C_3_HF_6_O^+^ have comparatively weak signals at all *E*/*N* values investigated, as highlighted in Figure 1. (See also Table 1 provided in the Supporting Information, which gives the product ion branching percentages for the six product ions at three of the *E*/*N* values; 70 Td, 140 and 200 Td.)

Given that the recombination energy of O_2_^+•^ is 12.07 eV,^36^ nondissociative charge transfer to HFIP leading to the formation of the transient ion C_3_H_2_F_6_O^+•^ and O_2_ is exoergic by 0.13 eV (12.5 kJ mol^–1^), assuming that O_2_^+•^ is not suprathermal. However, the precursor ion is not observed because it rapidly dissociates, initially to C_2_H_2_F_3_O^+^ or CF_3_^+^ through elimination of CF_3_^•^ or C_2_H_2_F_3_O^•^, respectively, from C_3_H_2_F_6_O^+•^. A subsequent elimination of HF from C_2_H_2_F_3_O^+^ leads to C_2_HF_2_O^+^, which in turn results in CHF_2_^+^ following a further elimination, in this case CO. (Figure S1 schematically summarizes the reaction pathways.) Using the GC-qToF-MS, we obtained the high mass resolving power 70 eV electron ionization mass spectrum (EI-MS) of HFIP (see Figure S2), which confirms that the four product ions above are correctly associated with a pathway that involves the removal of an electron from HFIP.

The other two product ions are observed with small branching percentages. These are C_3_HF_6_O^+^ (4% at 70 Td) and C_3_F_5_O^+^ (1% at 70 Td). Given that these are not observed in the EI-MS of HFIP, it is safe to conclude that they are not produced via a charge transfer channel. Instead, the proposed pathway leading to C_3_HF_6_O^+^ is via hydride anion (H^–^) abstraction from HFIP to O_2_^+•^. Once formed, a subsequent elimination of hydrofluoric acid (HF) from C_3_HF_6_O^+^ results in the only other product ion resulting from this channel, C_3_F_5_O^+^ (see Figure S1).

Of the three dominant product ions resulting from the charge transfer channel, namely CHF_2_^+^, CF_3_^+^, and C_2_H_2_F_3_O^+^, only C_2_H_2_F_3_O^+^ (at *m*/*z* 99.006) can be used in SRI-ToF-MS with any certainty to monitor HFIP in the exhaled breath of patients because (and as mentioned earlier) CHF_2_^+^ and CF_3_^+^ are also significant product ions resulting from the reaction of O_2_^+•^ with sevoflurane. (Note that although the sevoflurane product ion branching percentage for CF_3_^+^ is low, being approximately 0.1% at 113 Td and rising to about 4% at 200 Td,^6^ the significantly higher concentrations of sevoflurane compared to those associated with HFIP in exhaled breath mean that the contribution to the CF_3_^+^ signal intensity from sevoflurane swamps that resulting from HFIP.) Even using C_2_H_2_F_3_O^+^ as the probe ion for HFIP has some complications because measurements of samples containing large volume mixing ratios of sevoflurane (500 ppm_v_) showed that this ion is also a minor product ion resulting from sevoflurane’s reaction with O_2_^+•^, although having a branching percentage much less than CF_3_^+^ at all *E*/*N* values. However, for the HFIP measurements, we have determined that the contribution of sevoflurane to the ion signal intensity at *m*/*z* 99.006 for 113 Td is 1.7% and for 147 Td is 2.6% and when using the Nafion tube. Hence, the sevoflurane contribution to the signal intensity at *m*/*z* 99.006, although negligible, can be easily accounted for.

**Figure 2:**
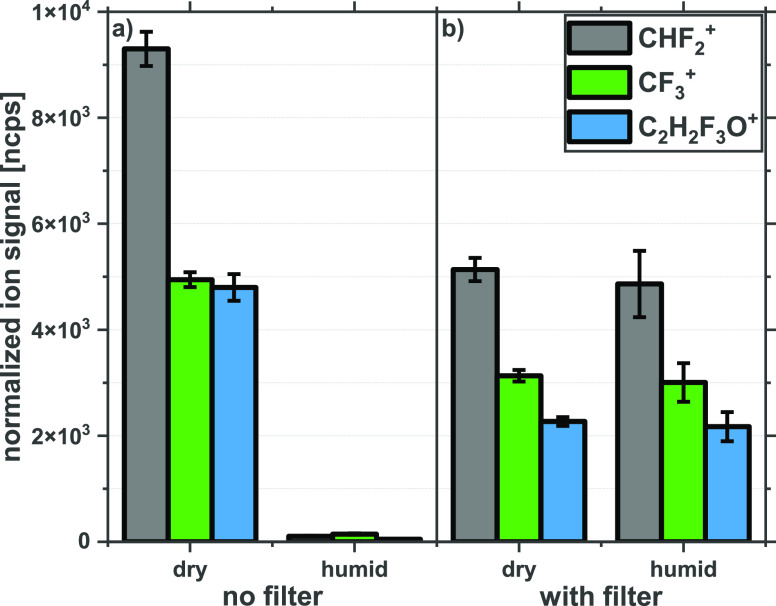
Comparison of the signals for the major product ions CHF_2_^+^, CF_3_^+^, and C_2_H_2_F_3_O^+^ in normalized counts per second (ncps) resulting from the reaction of O_2_^+•^ with HFIP. Product ion signals are recorded without and with a Nafion filter under both *dry* and *humid* nitrogen gas sample flows containing 1.05 ppm_v_ HFIP. The error bars represent one standard deviation. The PTR-TOF 6000 X2 was operated in DC-mode at a reduced electric field (*E*/*N*) value of 113 Td.

All of the product ions resulting from the reaction of O_2_^+•^ with HFIP are found to react efficiently with water vapor present in the drift tube. This will lead to a significant decrease in their intensities and hence a major loss in analytical sensitivity when it comes to applications involving the analysis of *humid* samples such as exhaled breath. To illustrate this, Figure 2a shows the normalized ion signal for the three dominant product ions CHF_2_^+^, CF_3_^+^, and C_2_H_2_F_3_O^+^, measured under *dry* and *humid* drift tube conditions. A HFIP volume mixing ratio of 1.05 ppm_v_ was used in these measurements, present in either *dry* (left-hand side of the Figure 2a) or *humid* (right-hand side of Figure 2a) nitrogen gas. Figure 2a clearly demonstrates that in going from a *dry* to a *humid* inlet carrier gas, the intensities of all three product ions are drastically reduced. This is significant that below a HFIP volume mixing ratio of about 500 ppb_v_ it becomes impossible to monitor the molecule with any assurance. Thus, the humidity in the drift tube of a SRI-ToF-MS will significantly affect the product ion distributions recorded and thereby severely limit the applications of SRI-ToF-MS for the detection and monitoring of HFIP in exhaled breath samples unless action is taken to reduce the humidity of the breath samples prior to their introduction into the drift tube.

With further reference to humidity effects, Figure 1a shows that the total product ion intensity is lowest at the lowest *E*/*N* value used, and increases as *E*/*N* increases until it reaches a maximum at about 160 Td, after which it declines with further increases in *E*/*N*. This type of product ion intensity behavior has been observed before in other PTR/SRI-ToF-MS studies when the primary product ions resulting from a compound react with water that is present in the drift tube.^5,6,18,37^ Although we operated the drift tube under what is defined as *dry* conditions to record the data represented in Figure 1a, water vapor is always present in the drift tube, as is apparent from reagent ions H_3_O^+^ and H_3_O^+^·H_2_O being observed. Even when using *dry* nitrogen carrier gas, there is sufficient water in the drift tube for the product ions signals to be reduced (see section 3.4.2 below). Furthermore, with decreasing *E*/*N*, the product ion signals will become more reduced owing to the increase in the reaction time for secondary processes with water vapor to occur. To confirm this, we undertook one further study whereby we flushed the hollow cathode and drift tube with *dry* nitrogen for 2 days and then we undertook two measurements directly one after the other, with the first using *dry* nitrogen and then the second with nitrogen at 5% rH. The product ion signals (ncps) and their branching percentages as a function of *E*/*N* under these two conditions are shown in the Supporting Information, Figure S3. Comparison of the results obtained using *dry* nitrogen carrier gas, Figure S3a, with those obtained using 5% rH nitrogen carrier gas, Figure S3b, confirms that the presence of water in the drift tube results in the observed product ion signals’ behavior.

### 3.4 Improving Analytical Sensitivity of the PTR 6000 X2 to HFIP

#### 3.4.1 Use of the Ion Funnel (RF-Mode)

Given the much lower concentrations of HFIP that will be found in exhaled breath, compared to those of sevoflurane, it is useful for breath applications to maximize the analytical sensitivity of the PTR 6000 X2 to detect HFIP. Hence, the operational parameters of the PTR 6000 X2 were optimized for the analysis of breath samples by employing the instrument’s ion funnel (i.e., operating the drift tube in RF-mode).

For the PTR 6000 X2 operating in DC-mode, C_2_H_2_F_3_O^+^ has its maximum intensity at an *E*/*N* value of 113 Td. Therefore, in RF-mode, the standard part of the drift tube was maintained at that reduced electric field value. The ion funnel was then turned on and tuned to obtain the highest possible sensitivity by maximizing the product ion signals, while maintaining a similar product ion distribution to that obtained in DC-mode at 113 Td. Under *dry* drift tube operating conditions, a significant increase in all of the HFIP product ion signals was obtained, while essentially maintaining the fragmentation pattern obtained in DC mode (see Figure S4). The increase in ion intensities was particularly apparent for CHF_2_^+^, CF_3_^+^, and C_2_H_2_F_3_O^+^, with their ion yields having all increased by approximately a factor of 5 upon the application of the ion funnel.

#### 3.4.2 Use of a Nafion Membrane to Reduce the Humidity of a Sample Gas Prior to Its Introduction into the Reaction Region (Drift Tube) of a PTR/SRI-ToF Mass Spectrometer

The effects on the product ion signals following the insertion of a Nafion tube into the sample inlet line under *dry* and *humid* nitrogen carrier gas flows are illustrated in Figure 2b. Using *dry* nitrogen gas flows, so that we are operating the drift tube under *dry* conditions, it can be seen that all of the product ions are reduced in intensity by approximately 50% compared to the case when no Nafion tube was used. This means that in addition to water loss, approximately 50% of the HFIP is also lost through the Nafion membrane during its flow through the tube. (Compare the dry nitrogen flow results for no Nafion (Figure 2a with Nafion Figure 2b.)

For a *humid* nitrogen gas carrier flow, Figure 2b shows that within experimental error the product ion signals are the same as those recorded with the *dry* nitrogen gas flow, meaning that most, if not all, of the water vapor has been removed by the Nafion. The important conclusion is that for a *humid* carrier gas containing trace levels of HFIP that flows through the Nafion tube prior to it being introduced into the drift tube, the loss of product ions resulting from the reduction in the HFIP volume mixing ratio is far less than that associated with the secondary reactions with water present in the drift tube. Effectively, the Nafion tube ensures the drift tube is operating under *dry* operating conditions even when a *humid* carrier gas flow is used or a *humid* sample is being analyzed. To illustrate the latter point practically, in the next section analytical measurements of *humid* exhaled breath samples containing HFIP are presented.

### 3.5 Analysis of Breath Samples Taken from a Volunteer Patient Following Surgery for which Sevoflurane was used as the Inhalation Anesthetic

**Figure 3:**
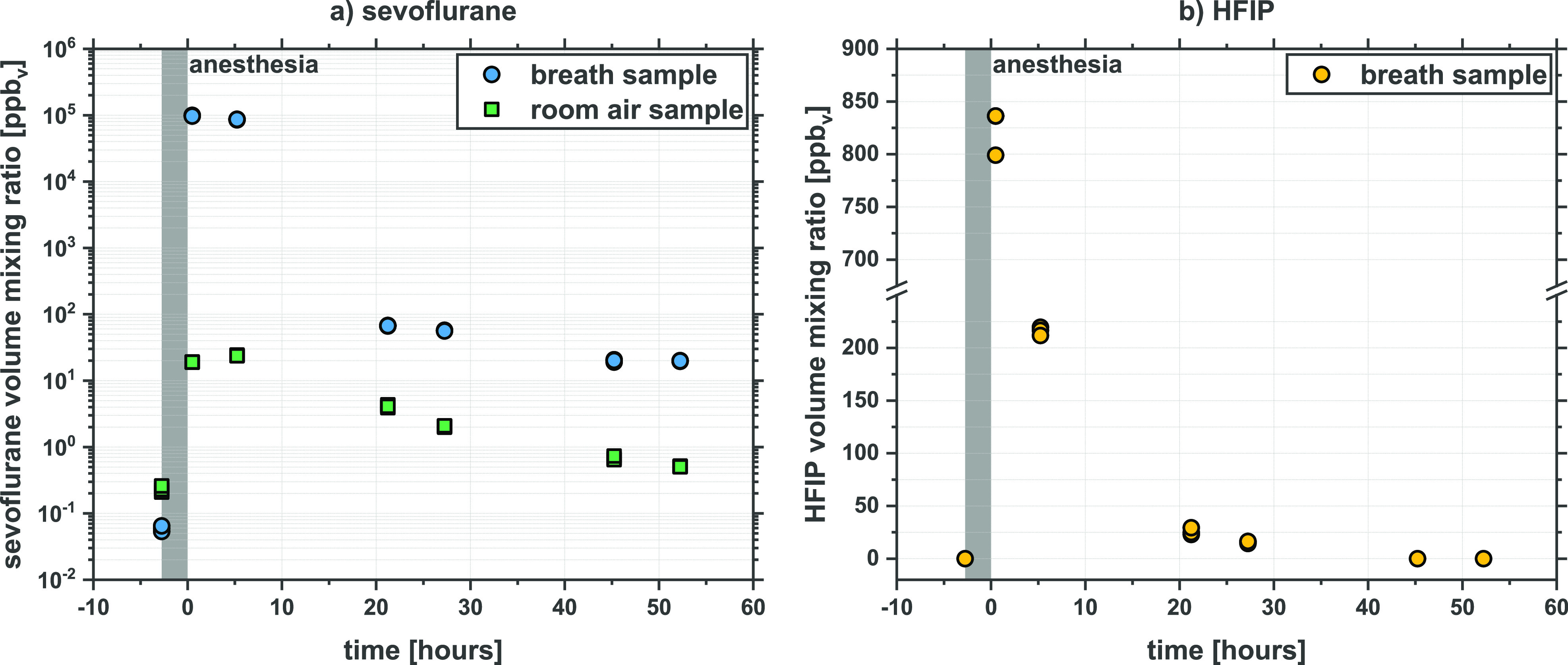
Volume mixing ratios of (a) sevoflurane (measured in DC-mode at an E/N value of 147 Td and using CHF_2_^+^ as the product ion marker) and (b) HFIP (measured in RF-mode and undiluted (see text)) in the exhaled breath of a volunteer patient following the end of anesthesia (*t* = 0 h). The Nafion tube was only used for the HFIP measurements. For sevoflurane, the Nafion tube was not needed because of the heavy dilution required to eliminate saturation of the product ion signals, which in turn eliminated the effects of humidity. Prior to surgery and at 45 and 52 h no HFIP was detected in the breath samples. In addition, all of the room samples had no HFIP at detectable levels.

As mentioned earlier, the much higher concentrations of sevoflurane compared to those of HFIP in the exhaled breath of patients exclude the use of both CHF_2_^+^ and CF_3_^+^ to monitor and quantify HFIP. However, C_2_H_2_F_3_O^+^ can be used as a marker for HFIP. Figure 3a presents the volume mixing ratios of sevoflurane in exhaled breath as well as in the room air, determined from the product ion CHF_2_^+^ at 147 Td in DC-mode (the contribution to the intensity of that product ion from HFIP being negligible), and (b) breath HFIP, determined from C_2_H_2_F_3_O^+^ at 113 Td and at 147 Td (for reasons given in section 2.7) in RF-mode as a function of time before and following the end of anesthesia.

Volume mixing ratios of sevoflurane found in the room air are much lower than those found in the breath samples. The volume mixing ratios of HFIP in the room air are below the limit of detection of our method and are therefore not included in Figure 3b, i.e., background levels of HFIP in the hospital environment are negligible compared to sevoflurane. The sevoflurane breath temporal profile behaves in a similar way to the earlier GC-MS study^3^ and our earlier SRI-ToF-MS study.^6^ Before surgery, the volume mixing ratio of sevoflurane is below the background levels measured in the hospital environment. The following data after surgery show significantly increased sevoflurane concentrations. Even 52 h after surgery, the breath levels are still higher compared to background levels. The shape of the HFIP breath profile is in good agreement to the one observed for sevoflurane. Starting from 0 ppb_v_ HFIP before surgery (i.e., no detectable HFIP in the breath and room samples), the concentration of HFIP is at approximately 830 ppb_v_ in exhaled breath in the first measurement taken directly after the surgical procedure. After 45 h the HFIP levels are below our limit of detection, and hence are represented as 0 ppb_v_ in the figure.

## 4 Discussion

Although there have been a number of soft chemical ionization mass spectrometric studies of volatile anesthetics in terms of fundamental ion molecule chemistry processes^5−8,38^ and for exhaled breath analysis,^9−11,39−43^ to our knowledge, none has investigated any associated volatile metabolites in any detail. In this study, we have investigated the chemical ionization of the metabolite of the inhalation anesthetic sevoflurane, namely HFIP with H_3_O^+^, NO^+^, and O_2_^+•^, as the reagent ions. In summary, we have reported for the first time PTR/SRI-ToF-MS investigations of HFIP using H_3_O^+^, NO^+^, and O_2_^+•^ as the reagent ions as a function of the drift tube’s reduced electric field (*E*/*N*) and the dependence of the product ion signals on the humidity of the gas in the drift tube. Only O_2_^+•^ shows any significant bimolecular reaction with HFIP, with the dominant primary product ions resulting from an initial charge transfer process.

Importantly, this investigation has raised again the problems of associated secondary ion–molecule reaction processes with soft chemical ionization mass spectrometric techniques, namely in this case the reaction of the HFIP primary product ions with water vapor present in the drift tube. This potential limitation of PTR/SRI-ToF-MS (and of course other soft chemical ionization mass spectrometric instruments such as SIFT-MS) to analyze volatiles in *humid* gas samples, such as exhaled breath, either off-line or in real-time, has been raised in several of our other recent publications.^5−7^ To overcome this problem, in this study we have adapted a technique used occasionally in gas chromatography to reduce the water vapor contained in samples before entering the GC column. For our SRI-ToF-MS measurements, this involved flowing *humid* gas samples, containing trace quantities of volatile compounds to be analyzed, through a Nafion tube. The Nafion tube was inserted into another tube to which a counter flow of *dry* nitrogen was applied. In this study, we have demonstrated the successful application of the Nafion tube for the analysis of trace HFIP in both *humid* nitrogen carrier gases and postoperational exhaled breath samples of a patient who underwent major surgery, for which sevoflurane was used as the inhalation anesthetic.

Of note is that this study has highlighted how HFIP in *humid* breath samples can easily be detected using O_2_^+•^ as the reagent ion in SRI-ToF-MS measurements provided a Nafion tube is incorporated into the sample inlet line prior to their introduction into the drift tube. Although the Nafion tube protocol developed is promising for analyzing volatiles in *humid* samples, this technique has some limitations owing to the potential loss of a significant fraction of the volatiles of interest resulting from their permeation through the walls of the Nafion tube. To decide if there is any gain in using a Nafion tube, any volatile loss has to be balanced against the need to allow sufficient time for water to diffuse to the membrane’s surface.^26^ The extent of reduction in a volatile’s concentration entering the drift tube will depend on the temperature, length and internal diameter of the Nafion tube, the carrier gas flow rate, and on the volatile’s specific chemical properties. Under our operating conditions, the loss of HFIP through the Nafion tube was found to be approximately 50%. This loss of HFIP is far less than the percentage loss of the HFIP’s product ions reacting with water in the drift tube. Therefore, in this study the Nafion tube has been demonstrated to be a valuable asset when analyzing breath samples containing HFIP. For lower molecular weight polar oxygenated compounds whose product ions react with water, the membrane will be far more permeable,^26^ and separate studies will be required to determine if there are any benefits to be gained by using this technique.

Owing to clinical restrictions, we were unable to undertake real-time analysis of HFIP in exhaled breath. Instead we have provided an off-line proof-of-principle clinical program, which has allowed us to monitor the production and elimination of HFIP from the body of a patient who had undergone surgery. This off-line clinical study clearly demonstrates the potential of chemical ionization mass spectrometry for such measurements. In particular, the protocol we adopted to reduce significantly the humidity of exhaled breath samples entering the reaction drift tube region of the SRI-ToF-MS dramatically increased its analytical sensitivity toward detection of HFIP in exhaled breath when using O_2_^+•^ as the reagent ion.

Importantly, this study has demonstrated the potential consequences for the improved analytical sensitivity of other volatiles whose primary product ions react with water present in the drift (reaction) tube of an analytical device that is based on chemical ionization mass spectrometry. A caveat with respect to the use of a Nafion tube to reduce the water content of a sample that is to be analyzed is that the volatiles of interest should not themselves be significantly lost as they pass through the Nafion tube. We plan further studies of volatiles in *humid* samples and explore other potential applications, especially focusing on key endogenous and exogenous volatiles commonly found in exhaled breath.

## Data Availability

Please contact the corresponding author Florentin Weiss.

## Supporting Information Available

Observed product ions and their associated branching percentages resulting from the reaction of HFIP with O_2_^+•^ as measured in DC-mode at three *E*/*N* values; proposed reaction pathways leading to the observed product ions resulting from the reaction of O_2_^+•^ with HFIP; high mass resolving power 70 eV electron ionization GC-qToF-MS of HFIP, with key ions identified; product ion signals in normalized counts per second (ncps) and as branching percentages resulting from the reactions of O_2_^+•^ with HFIP; comparison of the ion signals of the six HFIP product ions with the drift tube of the SRI-ToF-MS being operated in DC-mode and RF-mode (PDF)
