# Supplementary material for: Detecting Hexafluoroisopropanol Using Soft Chemical Ionization Mass Spectrometry and Analytical Applications to Exhaled Breath
Source: J Am Soc Mass Spectrom. 2023 Mar 30;34(5):958–68. doi: 10.1021/jasms.3c00042 (PMC10161230; doi:10.1021/jasms.3c00042)

Supporting Information

Detecting hexafluoroisopropanol using soft chemical ionisation mass spectrometry and analytical applications to exhaled breath

Florentin Weiss [1*], Anesu Chawaguta [1], Matthias Tolpeit [2], Valeria Volk [2], Arne Schiller [1], Veronika Ruzsanyi [1], Petra Hillinger [2], Wolfgang Lederer [2], Tilmann D. Märk [3], and Chris A. Mayhew [1]
[1] Institute for Breath Research, Universität Innsbruck, Innrain 66, A-6020 Innsbruck, Austria
[2] Department of Anaesthesiology and Critical Care, Medical University of Innsbruck, Anichstraße 35, A-6020 Innsbruck, Austria
[3] Institute for Ion Physics and Applied Physics, Universität Innsbruck, Technikerstraße 25/3, A-6020 Innsbruck, Austria

Corresponding Author
* Florentin Weiss - Institute for Breath Research, University of Innsbruck, Innrain 66, A-6020 Innsbruck, Austria.
e-mail: florentin.weiss@uibk.ac.at

Supplementary Table 1
Observed product ions and their associated branching percentages resulting from the reaction of HFIP with $O_2^{+\bullet}$ as measured in DC-mode at three *E/N* values (70 Td, 140 Td and 200 Td). The measured and actual *m/z* values are listed along with the difference between the two (Δ*m/z*), which shows that there is a systematic shift of approximately +0.002 in the experimentally determined value compared to the actual value. The *m/z* values are given for the lightest isotopomer. Note that the percentages measured are specific to the humidity conditions under which the drift tube is operated. Changes in humidity slightly alters the product ion branching percentages owing to differences in the reactivity of the product ions with water (see figure S3).

Table 1

**Hexafluoroisopropanol Structure Molecular Formula (*m/z* lightest-isotopomer)**	**Product ions**	**Product ion branching percentages**			**measured *m/z***	**actual *m/z***	**Δ*m/z***
		**70 Td**	**140 Td**	**200 Td**			
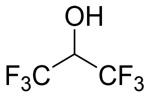	CHF_2^+	36	62	72	51.007	51.005	0.002
	CF_3^+	22	17	8	68.997	68.995	0.002
	$C_2HF_2O^+$	3	1	-	79.001	78.999	0.002
	$C_2H_2F_3O^+$	34	18	18	99.008	99.006	0.002
$C_3H_2F_6O$	$C_3F_5O^+$	1	-	-	146.989	146.987	0.002
(168.001)	$C_3HF_6O^+$	4	2	2	166.996	166.993	0.003

Supplementary Figures

Figure S1: The proposed reaction pathways leading to the observed product ions resulting from the reaction of $O_2^{+\bullet}$ with HFIP.

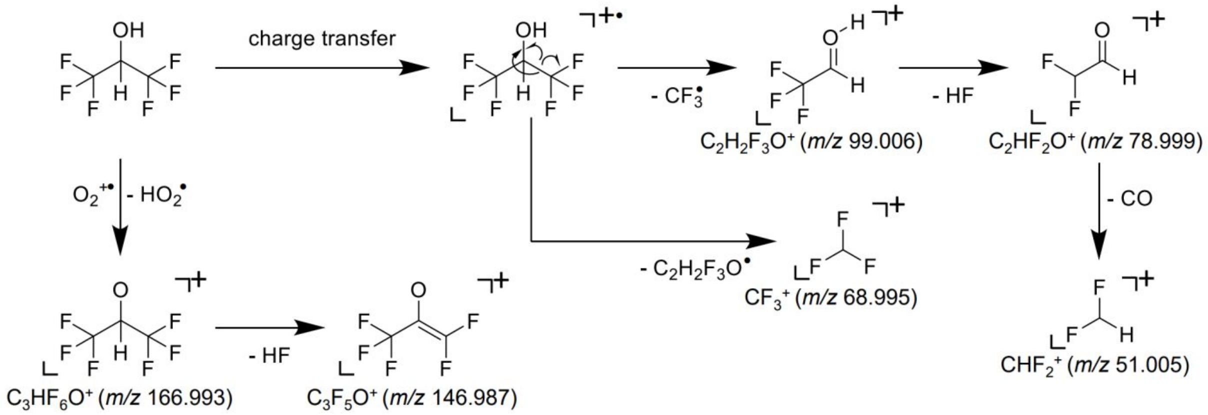

Figure S2: A high mass resolution 70 eV electron impact GC- qToF-MS of HFIP, with key ions identified.

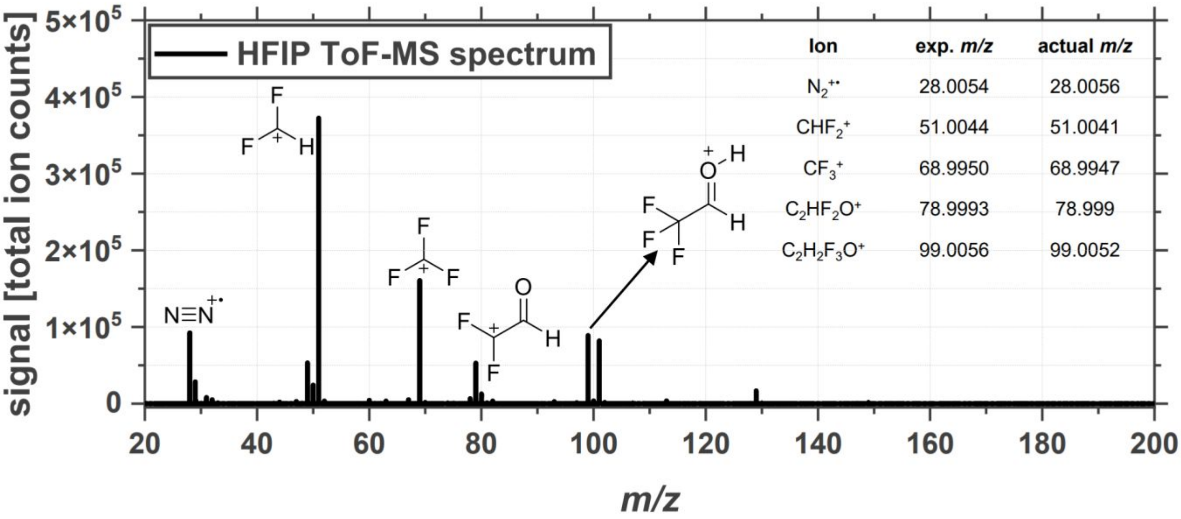

Figure S3: The product ion intensities in normalized counts per second (ncps) and as branching percentages resulting from the reactions of $O_2^{+\bullet}$ with HFIP (using a volume mixing ratio of 2.1 ppm_v) as a function of the reduced electric field (*E/N*) following two days of flushing the ionisation source and drift tube with *dry* nitrogen to reduce the water vapour concentration in the reaction region for (a) measurements with a *dry* nitrogen carrier gas (rH ~ 0%) that was immediately followed by (b) measurements with a nitrogen gas at a rH ~ 5%. Note the significant decrease in all product ion intensities over the whole of the *E/N* range investigated in going from a *dry* to a *humidified* nitrogen carrier gas.

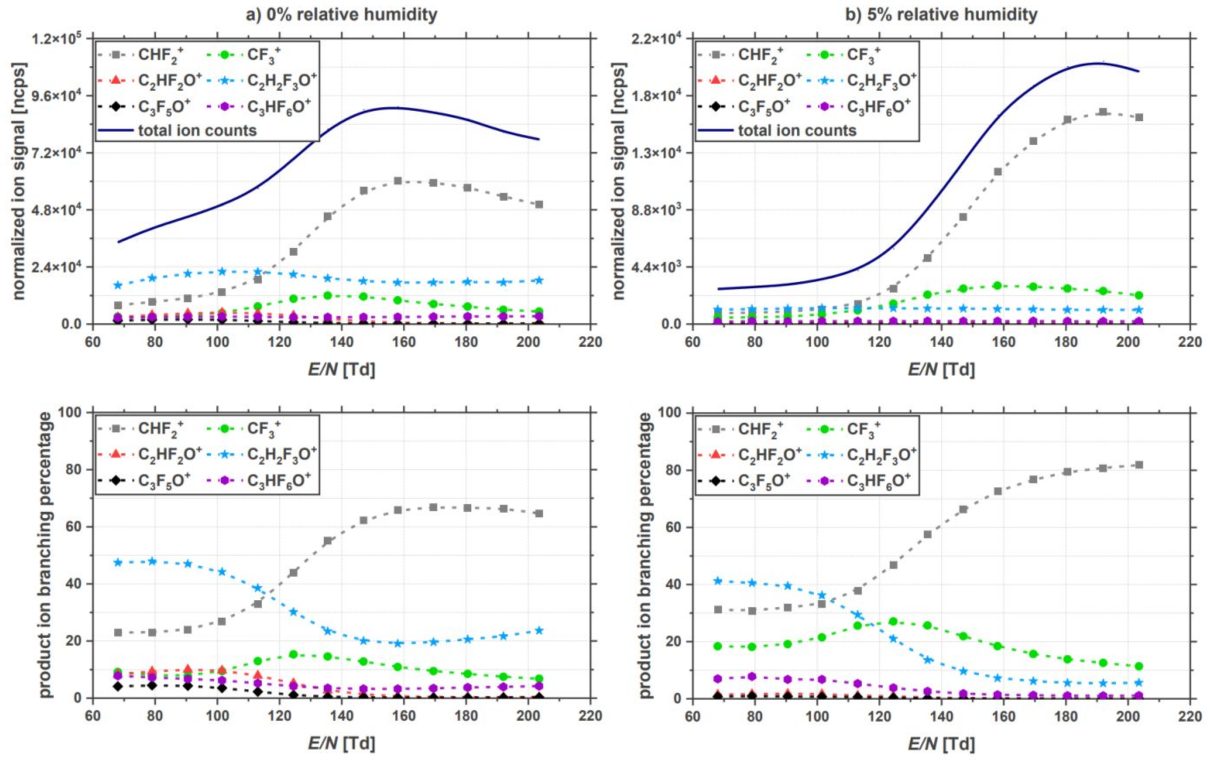

Figure S4: A comparison of the ion intensities of the six HFIP product ions with the drift tube of the SRI-ToF-MS operating in (a) DC-mode and (b) RF-mode to illustrate the gain in sensitivity. Both the DC and RF measurements were taken at an *E/N* value (*E/N* equivalent value in RF-mode) of 113 Td operating under *dry* drift tube conditions, using a gas standard containing 1.05 ppm_v of HFIP.

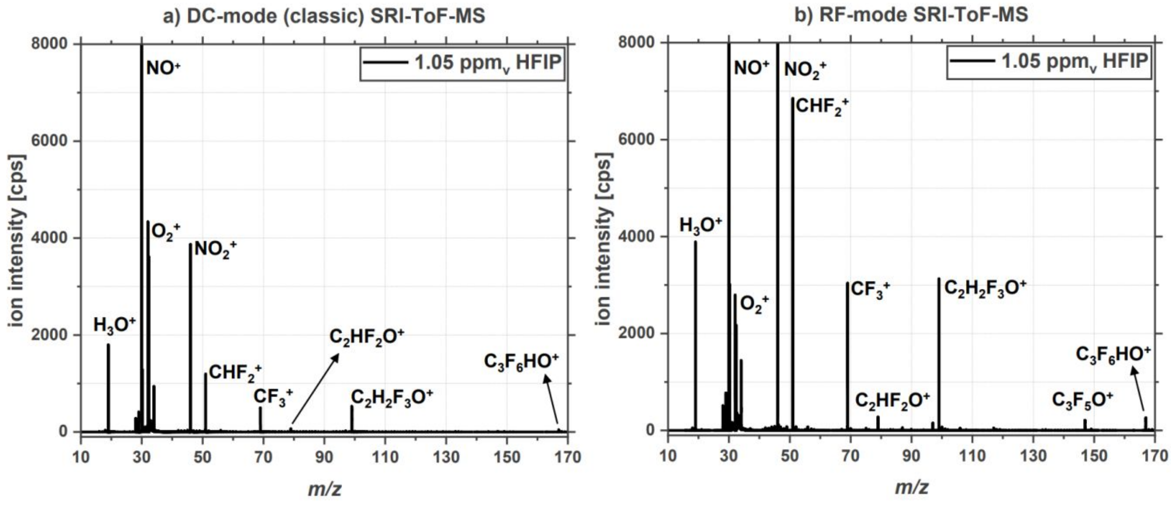

Supplement: Supplementary file 1 — js3c00042_si_001.pdf [file js3c00042_si_001.pdf]
